# Topical Uses of Iodine

**Published:** 1854-11

**Authors:** 


Topical Uses of Iodine.—The value of Iodine as a counter-irritant is year by year becoming more generally appreciated, and is yet much less so than it deserves. The iodine solution will probably, before long, entirely supersede mustard plasters, being at once more efficient, and much less disagreeable in its employment. The following notes on its applications will, perhaps, not be useless; they are the results of very extended observations in the hospitals generally, but more especially in those devoted to the treatment of diseases of the chest:—

1st. In the pleuritic stitches, or aching pains in the chest, so commonly recurrent in the course of phthisis, the iodine paint, applied over the affected spot, usually affords, without any expense to the vital powers, much more relief than either leeches, sinapisms or blisters. It may be used in almost all conditions of the system with perfect safety.

2nd. In cases of aphonia or hoarseness, depending on inflammatory thickening of the parts concerned in the production of voice, great benefit may be derived from painting the iodine over the front of the throat externally.

3rd. If the mucous lining of the fauces, etc., be thickened and congested, the solution may, without risk, be freely applied to the part itself.

4th. In the treatment of chronic enlargement of the tonsil, the application of iodine to the gland itself will sometimes effect a cure, but is much less generally efficient than constitutional treatment.

5th. In cases of chronic pleuritic effusion, or of consolidation of the lung, the solution should be painted over a large extent of the diseased side, and is of great service when the period for blistering or leeching has passed.

6th. Applied extensively over the belly, iodine is a useful counter irritant in the incipient stages of strumous peritonitis.

7th. In strumous ophthalmia, the application of the pharmacopœial tincture to the skin of the lids is often effectual in relieving intolerance of light; much benefit may also be derived from like practice in cases of granular lids. In both instances, frequent repetition is necessary.

8th. In all forms of periostitis, whether syphilitic, strumous, or the result of injury, iodine paint is invaluable.

9th. It is needless, perhaps, to mention the employment of iodine as a local application to bronchocele, to inflamed joints, and to the enlargements of the absorbent glands; with regard to the latter, a point is worthy of being borne in mind, to which Dr. Budd was, we believe, the first to direct attention, viz: the propriety of applying it to the skin beyond, and not over the affected gland, so as to allow of its being absorbed and taken through the gland in the course of the lymphatic circulation.

10th. Injections of iodine into the cavities of abscesses, glandular or otherwise, appear most frequently to produce good results, and to be unattended, except in very exceptional instances, by any risk. The theory of their use is, that the adhesive and not suppurative inflammations, as, for instance, in the radical cure of hydrocele.

11th. In cases of contracted cicatrices after burns, in which treatment by extension is adopted the application of iodine is of advantage in causing the absorption or softening down of the indurated structure. Some cases illustrative of this have recently been under care in the Middlesex Hospital. Care must be exercised, or ulceration may be caused.

12th. In cases in which the patient cannot be got to swallow medicine, as now and then happens in phagedæma of the throat, the specific influence of iodine may be induced by its endermic application, the best method being to paint over large surfaces of the skin the pharmacopœical tincture, choosing a different part each time.

The reason why, as a counter-irritant in all forms of chronic inflammation, iodine appears so superior to other applications, is doubtless to be found in the fact that it is capable of absorption, and may thus act beneficially in two distinct methods.

We have enumerated above some of the chief uses to which the iodine solution is daily put in the practice of the London Hospitals, but do not profess to have mentioned all. These are, however, enough, we think, to prove its right to a place on the dispensing table of every Medical practitioner.—*Med. Times and Gazette.*

